# Exploring the mechanical and morphological rationality of tree branch structure based on 3D point cloud analysis and the finite element method

**DOI:** 10.1038/s41598-022-08030-5

**Published:** 2022-03-08

**Authors:** Satoru Tsugawa, Kaname Teratsuji, Fumio Okura, Koji Noshita, Masaki Tateno, Jingyao Zhang, Taku Demura

**Affiliations:** 1 Department of Mechanical Engineering, Faculty of Systems Science and Technology, Akita Prefectural University, Akita, 015-0055 Japan; 2 Graduate School of Science and Technology, Nara Institute of Science and Technology, Nara, 630-0192 Japan; 3 Department of Architecture and Architectural Engineering, Kyoto University, Kyoto, 615-8540 Japan; 4 Graduate School of Information Science and Technology, Osaka University, Osaka, 565-0871 Japan; 5 Department of Biology, Kyushu University, Fukuoka, 819-0395 Japan; 6 Plant Frontier Research Center, Kyushu University, Fukuoka, 819-0395 Japan; 7 Nikko Botanical Garden, Department of Biological Sciences, Graduate School of Science, The University of Tokyo, Tochigi, 321-1435 Japan

**Keywords:** Plant sciences, Mathematics and computing

## Abstract

Trees are thought to have acquired a mechanically optimized shape through evolution, but a scientific methodology to investigate the mechanical rationality of tree morphology remains to be established. The aim of this study was to develop a new method for 3D reconstruction of actual tree shape and to establish a theoretical formulation for elucidating the structure and function of tree branches. We obtained 3D point cloud data of tree shape of Japanese zelkova (*Zelkova serrata*) and Japanese larch (*Larix kaempferi*) using the NavVis Lidar scanner, then applied a cylinder structure extraction from point cloud data with error estimation. We then formulated the mechanical stress of branches under gravity using the elastic theory, and performed finite element method simulations to evaluate the mechanical characteristics. Subsequently, we constructed a mechanics-based theoretical formulation of branch development that ensures constant bending stress produces various branching patterns depending on growth properties. The derived theory recapitulates the trade-off among branch growth anisotropy, stress-gravity length, and branch shape, which may open the quantitative way to evaluate mechanical and morphological rationality of tree branches.

## Introduction

Tree morphology is strongly influenced both by self-weight as it grows older and by environmental conditions such as wind^1,2^, snow^3^, and light^4^. Since the surrounding environment changes over time, trees have the potential to dynamically optimize their shape to deal with mechanical stress. Similar to Wolff’s law, where mechanical feedback reinforces bone density^5^, trees may reinforce their bodies in response to mechanical stress or loading, as exemplified by trunk bending and thigmotropism^6^. Furthermore, tree shape also depends on recovery from traumas, such as branch and trunk breaks^7^, hollow trunk^8^, wood decay^9^, cracks^6^, trimming^10^, and self-pruning^11,12^. Thus, tree shape is thought to be determined simultaneously by environmental responses and by self-optimization. However, a quantitative evaluation of such mechanisms has yet to be conducted, mainly because 3D tree morphology is geometrically complex and technically hard to quantify.

With emerging developments in computational technologies, the speed and accuracy of 3D laser scanners to obtain detailed point cloud data have improved^13^. 3D reconstruction of the skeletal structure of bare trees (i.e., lacking foliage) from 3D scans has been well studied using skeletonization techniques^14–16^, which can recover 3D structures representing actual tree shapes. However, for heterogeneous or incomplete 3D scans, it is better to fit the data to cylindrical structures rather than conducting naive skeletonization because the cylindrical fitting can be done only with a local heterogeneous or incomplete point cloud^17^. Cylinder-based approaches are often used in engineering^18–20^ and in architecture^21,22^, and a recent method for tree skeletal extraction using cylinder fitting shows promising results for heterogeneous point clouds^23^.

Leveraging the recent growth of 3D reconstruction methods to recover accurate tree structures from 3D point clouds, we aimed to unveil the relationship between the morphological and mechanical properties of trees. First, we hypothesized that the branch-growing process is mechanically unstable in terms of compressive and tensile stresses, and verified it by performing mechanical simulations using the finite element method, which is a similar approach to the recent works^24,25^. Using the mechanical results, we constructed a theoretical formulation of tree branch depending on mechanics, shape, and growth based on the axiom of constant stress^6,26^. The basic idea behind our modeling is that the tree branch experiences mechanical constraints, which affect its shape and/or growth. Information on this type of constraint and the interactions among mechanics, shape, and growth, may provide a solid platform for investigating the mechanical and morphological rationality of tree shape.

This paper is organized as follows. As examples of different types of tree shapes, we have selectively chosen a cone-shaped Japanese zelkova (*Zelkova serrata*) and a spreading-shaped Japanese larch (*Larix kaempferi*). The structures (skeletons with branch thickness) of the two tree shapes were extracted using a cylinder-based 3D reconstruction method. Subsequently, the statistical distributions of the radius, branch-opening angle, and branch-rotating angle of the cylinders (branch axes) were obtained. Using the morphological features, we used the finite element method to detect mechanical stress depending on the branch inclination angle, branch length, and branch radius. Consistent with the stress distribution, we also constructed a mechanics-based theoretical formulation of branching structure to demonstrate the possibility that the tree branch shape may result from the axiom of constant bending stress during development. Finally, we discuss some implications of our findings and possible future directions.

## Results

### Cylinder-based structure extraction reveals the detailed morphology of Japanese zelkova and Japanese larch

**Figure 1:**
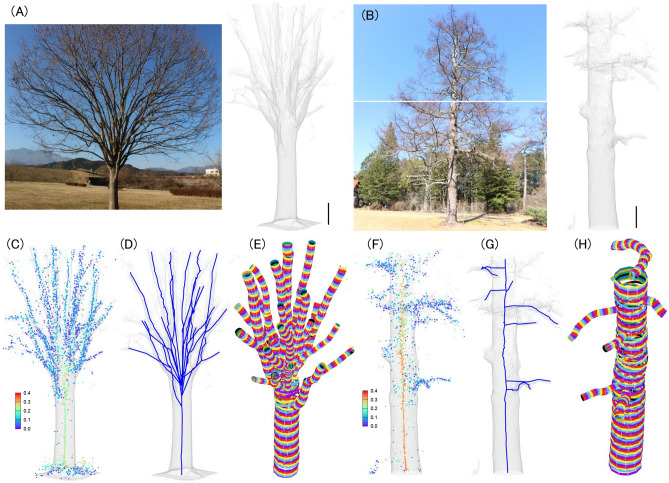
3D reconstruction of tree shapes. (**A**) Japanese zelkova. Scale bar is 50 cm. Photograph was taken by Kaname Teratsuji (Kyoto Univ.). (**B**) Japanese larch*.* Scale bar is 50 cm. Photograph was taken by Keiji Suzuki (image_technology_room, IIS, Univ. Tokyo). (**C**, **F**) Extracted centers of local cylinder structures for Japanese zelkova (**C**) and for Japanese larch (**F**). The color shows the radius of local circle. (**D**, **G**) Extracted skeletons derived from the EMST algorithm for Japanese zelkova (**D**) and for Japanese larch (**G**). (**E**, **H**) Extracted cylinders for Japanese zelkova (**E**) and for Japanese larch (**H**).

Figure 1A and B show the 3D point cloud data of Japanese zelkova and Japanese larch obtained by consecutive scans of the laser scanner (see Methods in details). From the point clouds, we extracted the skeletal structure as well as the branch thickness (Methods). Figure 1C and F depict the center of the cylinders colored by their radii (i.e., branch thickness). Figure 1D and G show the skeletal structure estimated by the Euclidean minimum spanning tree (EMST) algorithm (see Methods) and Fig. 1E and H render the extracted cylinders with their thickness. From these results, we could visually confirm that the detailed morphology of target trees was successfully extracted from 3D point clouds.

### The branch angles are different between Japanese zelkova and Japanese larch

**Figure 2:**
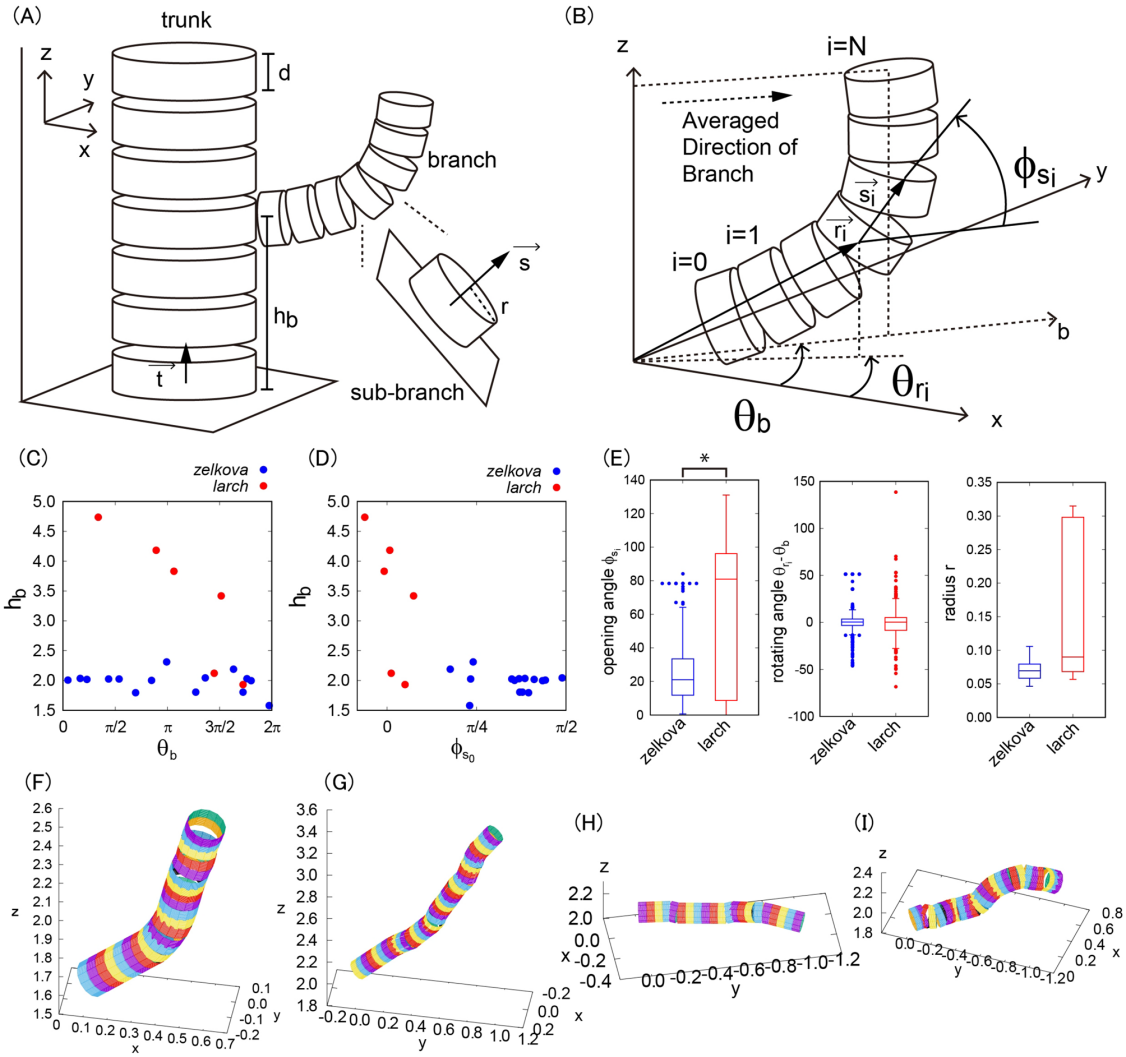
Quantification of some characteristic features of branching pattern. (**A**) Schematic illustration of a trunk, branch, and sub-branch. (**B**) Definition of averaged direction of branch and the rotating and opening angles of sub-branch. (**C**) Height of the branch base (m) as a function of rotating angle of branch (rad.). (**D**) Height of branch base (unit m) as a function of opening angle of branch (unit rad.). (**E**) Boxplots of the distributions of the opening angles (deg.), rotating angles (deg.) and radii (m) of all the sub-branches. The index * means the statistical difference with student’s t-test with *p* < 0.001. (**F**, **G**) Examples of Japanese zelkova branches. (**H**, **I**) Examples of Japanese larch branches.

Based on the extracted cylinder structure, we quantified some characteristic features of tree branches. Here, the whole tree structure was not examined because of sparse data points at the tips of the branches. Instead, we quantified detailed morphology in the range x: [−1 : 1 ], y: [−1 : 1 ], and z: [ 0 : 5 ] (m) with the origin of coordinates as the base of the trunk. For the following discussions, we named the vectors along the cylinder centers as $\overrightarrow{t}$ for trunk and $\overrightarrow{s}$ for branch. More precisely, we named the whole structure of the branch as “branch” and the local structure of the branch as “sub-branch” (Fig. 2A). The discretization scale is denoted by $d$ and the height of the base of the branch as ${h}_{b}$. The radius of the local cylinder is defined as $r$. For this setting, the averaged direction of the branch (ADB; ${\theta }_{b}$) is defined as the direction in which the branch is inclined with the averaged angle of sub-branches. The angle on the xy-plane is named as “rotating angle” hereafter and $\theta =0$ was chosen from $\mathrm{x}>0$, $\mathrm{y}=0$ of the original coordinate system. The rotating angle of the sub-branch ${\theta }_{{r}_{i}}$ was then defined as the xy-angle of the sub-branch with the position vector of the base of the sub-branch $\overrightarrow{{r}_{i}}$, and the opening angle of the sub-branch ${\phi }_{{s}_{i}}$ was defined as the deflection angle (bz-angle) from the axis of ADB ($b$-axis) to the discretized vector along the local cylinder ${s}_{i}$ (Fig. 2B).

As a result, we found that the ADB is very different between Japanese zelkova and Japanese larch (Fig. 2C), where Japanese zelkova has almost uniformly distributed angles at around 2.0 m while Japanese larch has a similar uniform distribution with different height (1.8–5.0 m). This indicates that the relationship between ${\theta }_{b}$ and ${h}_{\mathrm{b}}$ differs between tree types. For the bz-angle of branching (Fig. 2D), Japanese zelkova has angles with a range [ $\uppi /4$ : $\uppi /2$ ] while Japanese larch has angles around 0 with different height (1.8–5.0 m). This indicates that the relationship between ${\phi }_{{s}_{0}}$ and ${h}_{\mathrm{b}}$ also differs between the two tree types.

To capture a general trend of distributions of the rotating and opening angles and radii of sub-branches, we plotted all the data with boxplot (Fig. 2E). The opening angle ${\phi }_{{s}_{i}}$ was significantly different with student’s t-test (*p* < 0.001) whereas the rotating angle and radius were not significantly different. For example, the trend of the branching pattern was inclined in Japanese zelkova with the range [ $\uppi /4$ : $\uppi /2$ ] (Fig. 2F and G) and that in Japanese larch was almost horizontally aligned (Fig. 2H and I).

From these results, we hypothesized that the trees have different mechanical strategies that determine their shapes. In order to confirm this, we analyzed the mechanics of sub-branch morphology.

### Mechanical stress of the sub-branch derived from its own weight depends on the inclination angle of the sub-branch

**Figure 3:**
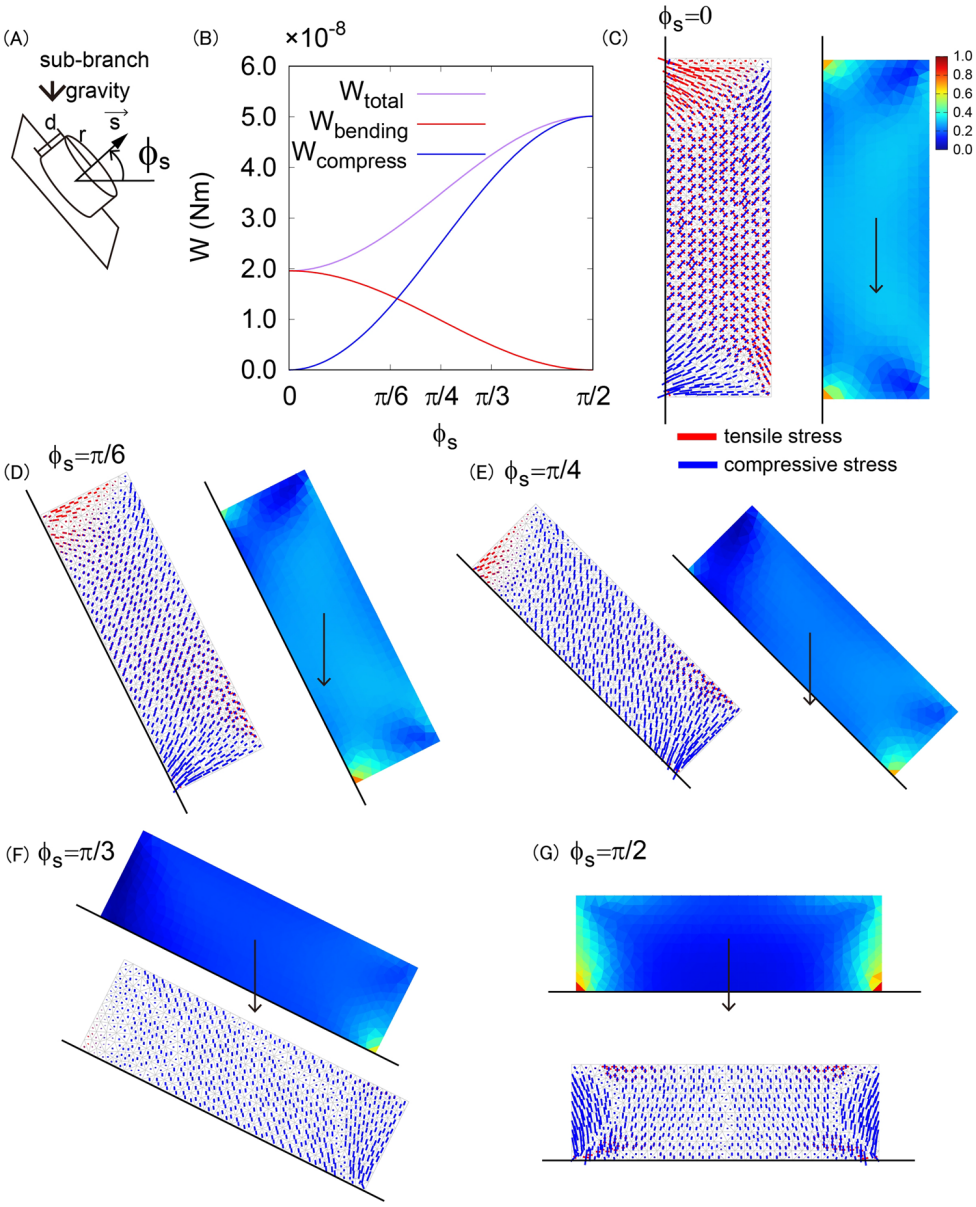
Mechanical stress derived from elastic theory and finite element method simulations depending on the sub-branch opening angle ${\phi }_{s}$. (**A**) Schematic illustration of sub-branch. (**B**) Theoretically estimated value of strain energy for bending (red), for compression (blue) and total of them (purple). (**C**–**G**) The mechanical stress vector field with tensile and compressive stress components (left or bottom) and von-mises stress distribution (right or top) are shown for ${\phi }_{\mathrm{s}}=0$ (**C**), ${\phi }_{\mathrm{s}}=\uppi /6$ (**D**), ${\phi }_{\mathrm{s}}=\uppi /4$ (**E**), ${\phi }_{\mathrm{s}}=\uppi /3$ (**F**), and ${\phi }_{\mathrm{s}}=\uppi /2$ (**G**).

To investigate the mechanics of sub-branch morphology, we performed the finite element method simulation (see Methods). We set up the 2D rectangular material under gravity with a fixed boundary condition on one side and a free boundary condition on the other side. With this setup, we can quantitatively evaluate the stress, strain and displacement of the cross section of the tree branch after the vertical self-weight was applied. We then systematically changed the opening angle of the sub-branch ${\phi }_{s}$ (Fig. 3A). In general, the material experiences three types of mechanical stress (bending stress, compressive or tensile stress, and shear stress^27^). Here, we only considered the mechanical stress of the sub-branch caused by its own weight. The shear stress is expected to be small compared with the other two (normal) stresses assuming the length of the sub-branch is long enough compared to the radius. The elastic theory provides us the strain energy derived from the bending stress with the angle ${\phi }_{s}$ as ${W}_{bending}=\frac{\pi {\left(\rho dg\right)}^{2}{d}^{3}}{2E}{\mathrm{cos}}^{2}{\phi }_{s}$ and that derived from the compressive stress as ${W}_{compress}=\frac{{\pi \left(\rho rg\right)}^{2}{d}^{3}}{2E}{\mathrm{sin}}^{2}{\phi }_{s}$ (Fig. 3B). To visualize these contributions, we plotted von Mises stress and the principal direction of stress as a function of the angle ${\phi }_{s}$ (Fig. 3C–G). As shown here, the sub-branch with angle ${\phi }_{s}=0$ similar to the Japanese larch type mainly suffers bending stress whereas the sub-branch with angle ${\phi }_{s}>\pi /4$ similar to the Japanese zelkova type suffers both bending and compressive stresses.

These are the fundamental mechanical differences depending on the branch inclination angle.

### Branch primary growth and secondary growth result in mechanical instability

**Figure 4:**
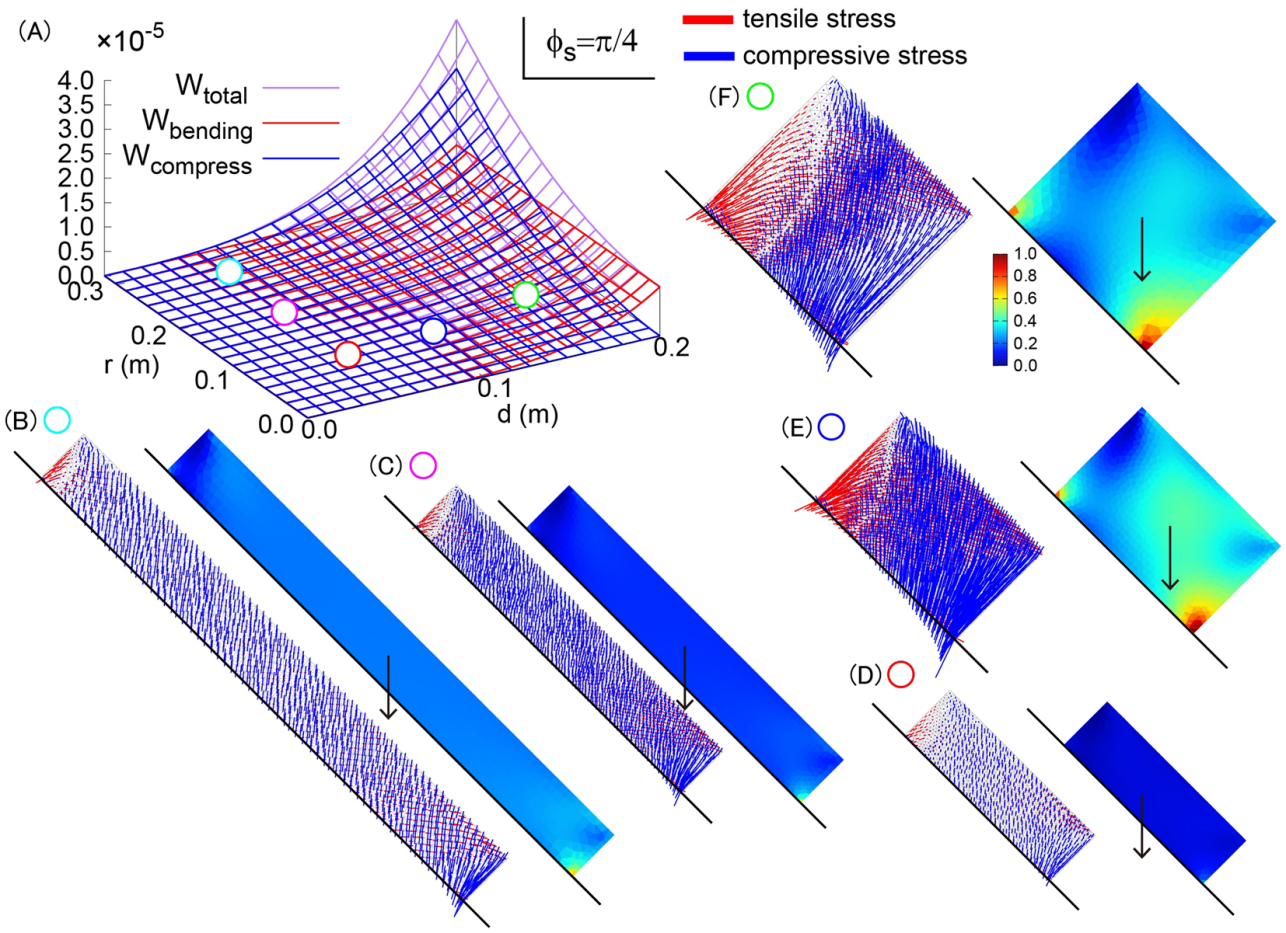
Mechanical stress derived from elastic theory and finite element method simulations depending on the length $d$ and radius $r$ of the sub-branch. (**A**) Theoretically estimated value of strain energy for bending (red), for compression (blue) and total of them (purple). (**B**–**F**) The mechanical stress vector field with tensile and compressive stress components (left) and von-mises stress distribution (right) are shown for $(d,r)=(\mathrm{0.05,0.24})$ (**B**), $(d,r)=(\mathrm{0.05,0.16})$ (**C**), $(d,r)=(\mathrm{0.05,0.08})$ (**D**), $(d,r)=(\mathrm{0.10,0.08})$ (**E**), and $(d,r)=(\mathrm{0.15,0.08})$ (**F**).

Next, we systematically changed the length and radius of the sub-branch $d$ and $r$ (Fig. 4). According to the strain energy formulation for bending stress and compressive stress of the sub-branch, it is expected that increases of both $d$ and $r$ result in increases in mechanical stresses. To confirm these hypotheses, we plotted von Mises stress and principal direction of stress as a function of $d$ and $r$ in Fig. 4. The sub-branch with large $d$ exhibits a larger magnitude of bending and compressive stresses. The sub-branch with large $r$ exhibits larger compressive stress whereas the bending stress was not influenced by this perturbation. These results indicate that the increase in length $d$ results in mechanical instability from bending and compressive stresses, and on the other hand, the increase in radius $r$ results in mechanical instability only from compressive stress, being consistent with the strain energy formulations.

To summarize the results, the branch primary growth with $d$ is a mechanically unstable process both in bending and compressive stresses, and the branch secondary growth with $r$ is a mechanically unstable process in compressive stress.

### Theoretical formulation of branch structure based on local stress relaxation by changing its growth direction

**Figure 5:**
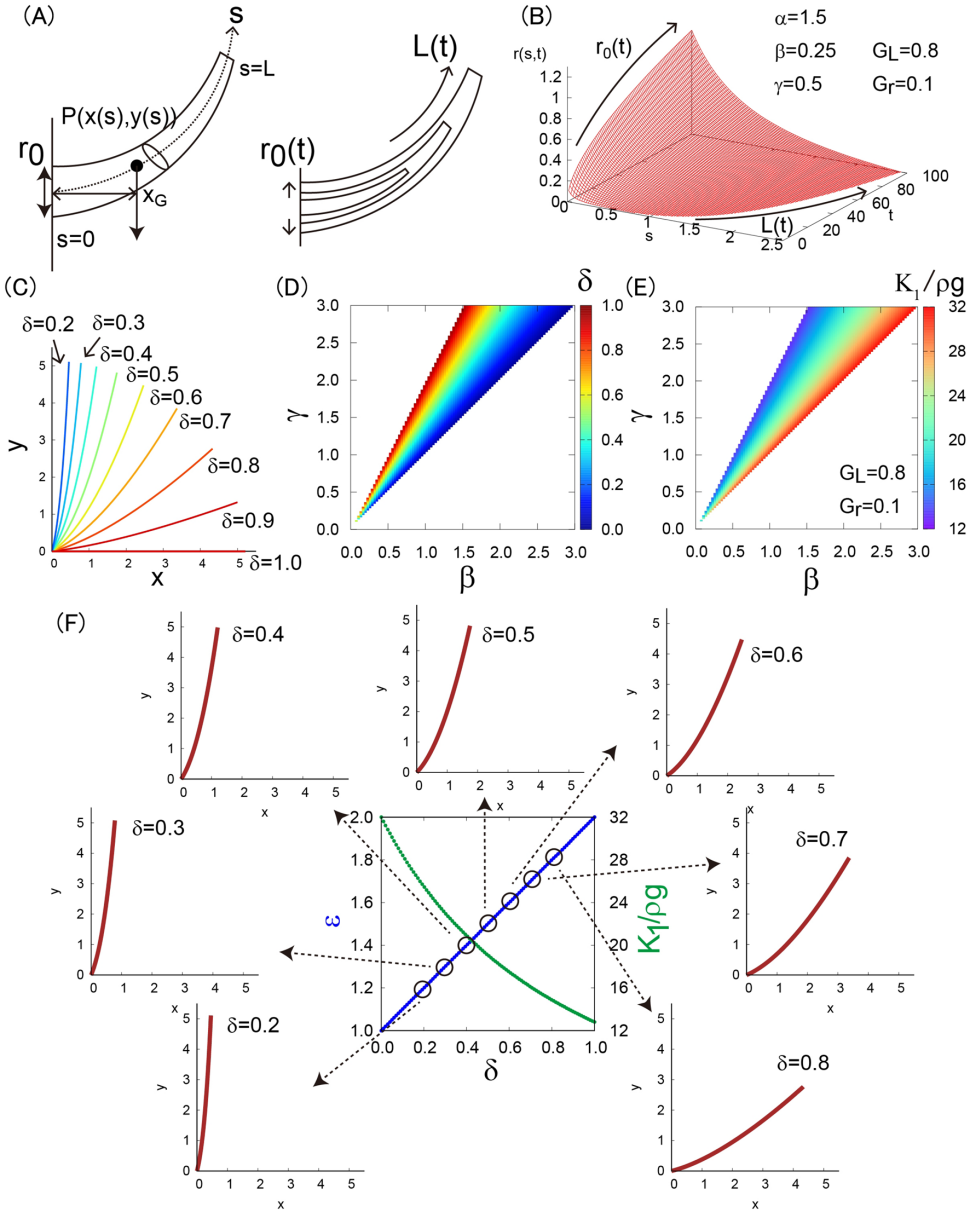
Theoretical formulation of branch structure. (**A**) Schematic illustration of the growing branch model. (**B**) A typical example of r(s,t). (**C**) Branch shape function. (**D**) The shape parameter $\updelta$ as functions of $\upbeta$ and $\upgamma$. (**E**) The mechanical parameter ${K}_{1}/\rho g$ as functions of $\upbeta$ and $\upgamma$. (**F**) Diagram of the trade-off between growth anisotropy $\upvarepsilon$ and stress-gravity length ${K}_{1}/\rho g$ as a function of the shape parameter $\updelta$.

These mechanical results described above demonstrate that the branch suffers more stress by increasing its length and radius. It means that the branches are inherently required to deal with this excess stress by growth in order to avoid a crack or, in the worst case, a break. We therefore hypothesized that the branch may avoid this mechanical instability by changing its direction of growth to locally relax the mechanical stress. For branches, the bending stress should be the dominant factor, so we only considered bending stress in the following formulation. To consider the hypothesis, we set up a virtual growing branch in length and in radius (Fig. 5A). By defining the curved coordinate along branch $s$ where $s=0$ at the branch base and $s=L$ at the branch tip, we defined the primary growth using $L(t)$ and the secondary growth using $r(s, t)$. At the branch base, we used the radius ${r}_{0}(t)=r(s=0, t)$. We assumed that the concentrated weight is applied at the barycenter of the branch with x-coordinate ${x}_{G}$. In the literature, this formulation is similar to the cost function of material building energy and bending strain energy of a 2D straight beam that is based on the axiom of constant bending stress^26^.

Depending on the input of the branch shape function $y=f(x)$ and growth properties of $L(t)$ and ${r}_{0}(t)$, we can evaluate the mechanical state associated with branch growth. For example, the spatio-temporal radius in a tapered case is defined as

$$r\left(s, t\right)={r}_{0}\left(t\right)\frac{{\left({r}_{0}\left(t\right)+s\right)}^{-\alpha }-{\left({r}_{0}\left(t\right)+L(t)\right)}^{-\alpha }}{{{r}_{0}\left(t\right)}^{-\alpha }-{\left({r}_{0}\left(t\right)+L(t)\right)}^{-\alpha }}.$$

where the primary growth is assumed as $L\left(t\right)={G}_{L}{t}^{\beta }$ and the secondary growth at the branch base is assumed as ${r}_{0}\left(t\right)={G}_{r}{t}^{\gamma }$. The branch tapered property from base to tip is denoted by the index $\alpha$. For the case with $\alpha =1.5, \beta =0.25, \gamma =0.5, {G}_{L}=0.8,$ and ${G}_{r}=0.1$, the spatio-temporal distribution of $r\left(s, t\right)$ can be evaluated (Fig. 5B). In the following formulation, we set the radius spatial distribution as a constant, i.e., $r\left(s\right)={r}_{0}$, because we did not obtain tapering information due to the restricted spatial scale.

To test the hypothesis, we assumed that the maximal bending stress at the branch base is constant during growth. The constant maximal bending stress can be formulated as

$$\tau \left(t\right)=\frac{w\left(t\right){x}_{G}\left(t\right)}{Z(t)}=\frac{4\rho g\int_{0}^{L(t)}r{\left(s,t\right)}^{2}ds}{{r}_{0}{\left(t\right)}^{3}}{x}_{G}\left(t\right)=const({K}_{1})$$

where the self-weight is $w\left(t\right)=\rho g\int_{0}^{L(t)}\pi {r\left(s,t\right)}^{2}ds$, and the section modulus is $Z\left(t\right)=\pi {r}_{0}{\left(t\right)}^{3}/4$. In the case of a non-tapered branch with $r\left(s,t\right)={r}_{0}(t)$,

$$\tau \left(t\right)=\frac{4\rho gL(t){x}_{G}\left(t\right)}{{r}_{0}(t)}=const({K}_{1}).$$

As a result, the x-coordinate at the barycenter ${x}_{G}\left(t\right)$ is given by,

$${x}_{G}\left(t\right)=\frac{{K}_{1}{r}_{0}(t)}{4\rho gL\left(t\right)}=\frac{{K}_{1}{G}_{r}}{4\rho g{G}_{L}}{t}^{\gamma -\beta }.$$

On the other hand, the index ${x}_{G}\left(t\right)$ as a geometric definition can be written as

$${x}_{G}\left(t\right)=\frac{\int_{0}^{L(t)}\pi {r}_{0}^{2}x(s)ds}{\int_{0}^{L(t)}\pi {r}_{0}^{2}ds}=\frac{\int_{0}^{L(t)}x\left(s\right)ds}{L\left(t\right)}.$$

Therefore, the governing equation of branch shape $x\left(s\right)$ can be given as,

$$\int_{0}^{L(t)}x\left(s\right)ds=\frac{{K}_{1}{G}_{r}}{4\rho g}{t}^{\gamma }.$$

In the simple case with $x\left(s\right)=s$ corresponding to $f\left(x\right)=0$^26^, the equation becomes $\frac{L{\left(t\right)}^{2}}{2}=\frac{{K}_{1}{G}_{r}}{4\rho g}{\mathrm{t}}^{\upgamma }$, which results in

$${G}_{L}^{2}=\frac{{K}_{1}{G}_{r}}{2\rho g},\quad 2\upbeta =\upgamma .$$

These are the governing growth constraints for a straight-shaped branch derived from constant bending stress during growth. For the general case with $x\left(s\right)={s}^{\updelta } (\updelta >0)$, the shape function can be expressed as, for example $f\left(x\right)={(x+1)}^{\frac{1}{\delta }}-(x+1)$ with $y\left(s\right)={({s}^{\delta }+1)}^{\frac{1}{\delta }}-({s}^{\delta }+1)$, and the equation becomes $\frac{L{\left(t\right)}^{\delta +1}}{\delta +1}=\frac{{K}_{1}{G}_{r}}{4\rho g}{\mathrm{t}}^{\upgamma }$. The growth constraints become

$$\frac{{K}_{1}}{\rho g}=\frac{4{G}_{L}^{\delta +1}}{{G}_{r}(\delta +1)},\updelta =\frac{\upgamma }{\upbeta }-1. (*)$$

To visualize the obtained result (*), we plotted the shape function with various values of $\delta$ (Fig. 5C). In this visualization, the branch length is fixed with a constant for comparison,

$$L\left({t}_{c}\right)=\underset{0}{\overset{{t}_{c}}{\int }}\sqrt{1+{\left(\frac{dy\left(s\right)}{dx(s)}\right)}^{2}}dx,$$

where ${t}_{c}=5.2 (\mathrm{a}.\mathrm{u}.)$. We also show the color plots of the index $\delta$ and the target stress-gravity length ${K}_{1}/\rho g$ as functions of $\beta$ and $\upgamma$ (Fig. 5D and E). These diagrams represent the growth constraints, which connect the branch shape, growth, and mechanics. Here, the index $\upvarepsilon =\upgamma /\beta$ indicates the growth anisotropy which is the ratio of the primary (axial) growth and secondary (radial) growth. The index ${K}_{1}$ indicates the target stress which is the expected stress from self-weight where the tree branch can keep the horizontal morphology if the target stress becomes low (with a light loading) whereas it cannot keep it if the target stress becomes high (with a heavy loading). Therefore, the index ${K}_{1}/\rho g$ indicates how the target stress contributes to the self-weight which can be selectable by tree, therefore we call it a mechanical strategy parameter. Finally, we evaluated the trade-off between growth (growth anisotropy $\upvarepsilon =\upgamma /\beta$) and mechanics (stress-gravity length ${K}_{1}/\rho g$) as a function of the shape parameter $\updelta$ (Fig. 5F). The meaning of the trade-off is revisited in the discussion section.

**Figure 6:**
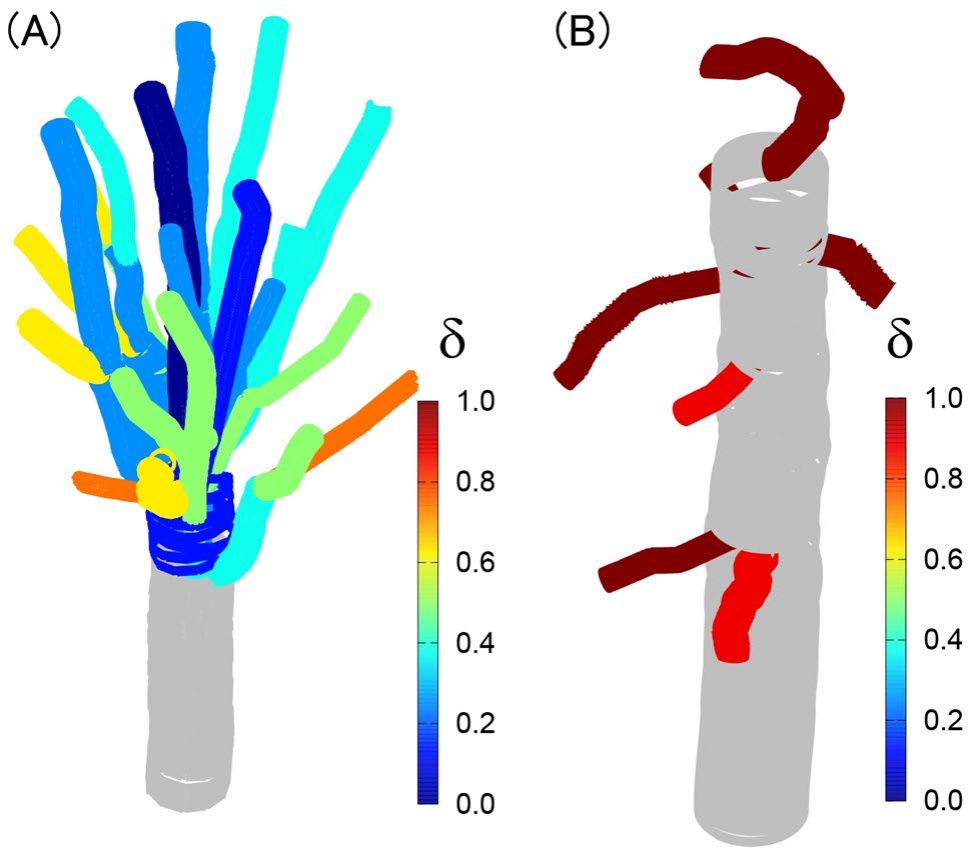
(**A**, **B**) Shape parameter $\updelta$ for each branch for Japanese zelkova (**A**) and for Japanese larch (**B**).

From the data analyses, the shape parameter for Japanese zelkova was $\updelta =0-0.7,$ i.e., the growth anisotropy was low and the target stress was high, and the shape parameter for Japanese larch was $\updelta =0.9\sim 1.0$, i.e., the growth anisotropy was high and the target stress was low (Fig. 6). These results mean that the growth-mechanics strategy differs between these two tree types, which determines their unique shapes.

Overall, we theoretically formulated the relationship between branch shape, growth, and mechanics based on the axiom of constant bending stress.

## Discussion

In this study, we evaluated the detailed morphology of Japanese zelkova and Japanese larch and constructed two mechanical hypotheses. The first hypothesis was that the trees have different mechanical strategies that determine their shapes; this was supported by the following two mechanical results: (1) The mechanical stress depends on the branch inclination angle. (2) Branch primary and secondary growth result in mechanical instability. These findings prompted us to consider the next hypothesis that the branch may avoid the mechanical instability by changing its growth direction to locally relax the mechanical stress. Based on the axiom of constant maximal bending stress discussed with a 2D straight beam^26^, we formulated the governing equation of curved branch shape, which recapitulates the trade-off among branch growth anisotropy, stress-gravity length, and branch shape.

What are the biological implications of the trade-off among growth, mechanics, and shape? The meaning of the trade-off between growth anisotropy and stress-gravity length depending on the branch shape (vertical or horizontal) is that the growth anisotropy increases and the branch stress level decreases as the branch angle inclines to be horizontal. More precisely, we found that there exists a growth-shape constraint ($\updelta =\frac{\upgamma }{\upbeta }-1$), i.e., a growth pattern realizing the relatively constant barycenter, and a mechanics-shape constraint ($\frac{{K}_{1}}{\rho g}=\frac{4{G}_{L}^{\delta +1}}{{G}_{r}(\delta +1)}$), i.e., maintaining the bending stress at the target which is often the upper limit of the bending stress. As stated in a previous study^26^, the branch shape may be constrained by mechanics through maximal bending stress. In addition to this finding, we explored the growth-shape constraint using a more general spatio-temporal branch shape, which covered shape (length and radius) and mechanical properties (bending and compressive stresses) at the sub-branch scale. This demonstrated that the growth, mechanics, and shape are highly constrained by each other. Furthermore, one can use these constraints to predict the growth or mechanical strategies of a tree branch only from branch shape.

Tree botanical studies are closely related to studies of evolution^27,28^. Generally, tree shape is classified into two categories. One is the excurrent shape with a cone-shaped morphology represented by Japanese larch, and the other is the decurrent shape with a spreading-shaped morphology represented by Japanese zelkova. Using the obtained growth-mechanics-shape constraint, the next challenge might be a growth or mechanical classification based on the actual tree point cloud data. In our measurements and estimations of mechanical properties, Japanese larch showed high growth anisotropy and low target stress, and Japanese zelkova showed low growth anisotropy and high target stress. This implies that Japanese larch has a higher potential to form wide branching patterns than Japanese zelkova. As many point cloud data will be obtained in the future, the data-model combined approach may become a simple and powerful analytical tool to study mechanical characteristics of woody plant due to changes in their morphological structure. It will also undoubtedly raise new questions in plant science, physics, information science, agriculture, and mechanical engineering.

## Methods

We acquired 3D point cloud data of the target trees with a laser scanner where the target trees were not foliaged in order to obtain the accurate branch structure. Inputting the point cloud data, we extracted the skeleton structure of the dominant branches using point cloud processing. The extracted structures were used for mechanical simulation by the finite element method.

### Point cloud data acquisition

The point cloud data for Japanese zelkova (*Zelkova serrata*) (Keyaki in Japanese) and Japanese larch (*Larix kaempferi*) (Karamatsu in Japanese) were obtained by a NavVis Lider scanner (M6 Trolley, NavVis, USA) at Nikko Botanical Garden (the Graduate School of Science, University of Tokyo; 1842 Hanaishi-cho, Nikko, 321–1435 Japan) and at Nikko Daiya River Park (844 Segawa, Nikko, 321–1263 Japan). The scanner was moved around the trees to capture the traveling distance of emitted lights between the observed point and the tree. We then aligned the captured 3D scans to create a single point cloud data using the software bundled with the scanner.

### Cylinder-based skeletal structure extraction

Given a point cloud, we extracted the skeletal structure of the target tree. Here, we describe the details of our algorithm, although it resembles the existing cylinder-based approach^23^.

Our method first finds the local axes (i.e., branch directions) from the tangent plane fit to the neighboring points. We then fit cylinders along the axes to represent local branches. We finally extracted the skeletal structure using the EMST algorithm. We describe the detail of each process below.

#### Local tangent plane fitting

We first found the local tangent plane of the randomly picked $i$-th point, ${p}_{i}=({x}_{i},{y}_{i},{z}_{i})$, based on the error between a plane and the data, i.e., ${E}_{i} ={\left(a{x}_{i}+b{y}_{i}+c-{z}_{i}\right)}^{2}$. A set of neighboring points of ${p}_{i}$ was selected as the points inside a sphere with radius ${r}_{n}$ centered by ${p}_{i}$. We empirically determined ${r}_{n}$ so that at least 15 points were contained in the sphere (see Figure S1 for details).

#### Local branch direction estimation

After fitting the local tangent plane, we estimated the local branch direction where ${p}_{i}$ belongs to, which will be the axis direction of the cylinder. Figure S2 illustrates a synthetic example. We set a certain threshold $\alpha {h}_{c}$ ($0<\alpha <1$) where the index ${h}_{c}$ is the maximal height of the points from the local tangent plane. We picked all the data points below the threshold and applied principal component analysis (PCA). By changing the parameter $\alpha$, the local axial data structure was extracted (see Figure S2B). In our experiment, we found $\alpha$ needs to be less than 0.7 to detect the orientation by PCA with our desired precision 0.1 (radian).

#### Local cylinder fitting

Once the axis was determined, we projected the points around ${p}_{i}$ on a plane perpendicular to the axis and fit a circle on the plane. To assess the reliability of the obtained circle, we applied this method with different $\alpha$, and found that less than 0.3–0.4 is needed to detect the precise circle with our desired precision of 0.1 (radian), as shown in Figure S3. Therefore, we used the parameter $\alpha =0.3$ to ensure $\alpha <0.7$ for the orientation detection mentioned above. After fitting the circle, we determined the corresponding cylinder so that it had the axis with estimated direction passing through the center of the estimated circle. We simultaneously yielded the local branch thickness as the radius of the estimated circle.

#### Skeleton extraction using Euclidean minimum spanning tree (EMST)

The basic idea of the EMST is that the skeletal structure of the tree shape can be described by the sum of the edge weights (e.g. Euclidean distance of the sub-branch length in our case) being as small as possible. Once we get the edge weights, the connected network in space can be reconstructed which is the description of the skeleton structure of the tree shape. In order to use EMST, we firstly avoid the large fitting error as shown in Fig. 1C and F. To decrease the outliers, we discarded the cylinders with a higher fitting error larger than using a certain error threshold (0.02 m in mean absolute error). After this process, we could obtain the skeletal structure with a few exceptions, those which were located outside the tree or missing branch points. We thus manually removed the outliers and inserted several missing points using MeshLab software. After these discarding processes, we applied the EMST algorithm to obtain the skeleton of the point cloud.

### Finite element method simulations

We built a mechanical model of branch structure based on finite element method simulations using FreeFem++^29^. Assuming the 2D solid rectangular material under gravity with a fixed boundary condition on one side and a free boundary condition on the other side, we modeled a cross section of the tree branch. We used 2D linear triangle elements in order to quantify the inner stress structure under gravity. The nodes and elements are generated such that the side of the triangle element becomes sufficiently smaller than 1/50 of the branch length. For the stress–strain relationship, we used the generalized Hooke’s law linking the stress tensor $\upsigma$ and the strain tensor $\upvarepsilon$ through the elasticity matrix,

$$\left(\genfrac{}{}{0pt}{}{{\sigma }_{xx}}{\begin{array}{c}{\sigma }_{yy}\\ {\sigma }_{xy}\end{array}}\right)=\left(\begin{array}{ccc}A& B& 0\\ B& A& 0\\ 0& 0& C\end{array}\right)\left(\genfrac{}{}{0pt}{}{{\varepsilon }_{xx}}{\begin{array}{c}{\varepsilon }_{yy}\\ {\varepsilon }_{xy}\end{array}}\right),$$

where $\mathrm{A}=\frac{\mathrm{E}}{1-{\upnu }^{2}}, B=\frac{\mathrm{E\nu }}{1-\upnu }, C=\frac{E}{1+\nu }$, E being the elastic modulus, and $\nu$ being Poisson’s ratio. In the simulation, $E$ and $\nu$ were set to be 10 GPa and 0.35, respectively. We quantified stress, strain, and displacement of the branch under the uniform self-weight condition. We changed the branch base angle and corresponding fixed boundary condition and changed the morphology (length and radius of the branch) and obtained the stress information inside the branch.

## Supplementary Information

Supplementary Legends.

Supplementary Figure 1.

Supplementary Figure 2.

Supplementary Figure 3.
