# Supplementary material for: Exploring the mechanical and morphological rationality of tree branch structure based on 3D point cloud analysis and the finite element method
Source: Sci Rep. 2022 Mar 8;12:4054. doi: 10.1038/s41598-022-08030-5 (PMC8904476; doi:10.1038/s41598-022-08030-5)
Supplement: Supplementary file 1 — Supplementary Legends. [file 41598_2022_8030_MOESM1_ESM.docx]

**Figure legends**

Fig.S1. Tests of fitting the local tangent plane. (A) Test data of a cylinder with radius 0.2 m (gray), point of interest (red), points for fitting of the local plane with the radius 0.05 m (blue), and the fitted plane (green). (B) Examples of the local tangent planes with point clouds of different densities. (C) Error estimation as a function of the number of points in the sphere.

Fig.S2. Orientation rearrangement by principal component analysis (PCA). (A) A local point cloud in test data with height $h_{c}$. We set the index $\alpha$ which defines the region of interest to detect the axis of the cylinder. (B) A few examples of the regions of interest depending on the index $\alpha$. The orientation of the detected axis of the cylinder is denoted by $\phi$. (C) Error estimation as a function of the index $\alpha$.

Fig.S3. Circle fitting perpendicular to the cylinder axis. (A, B) Detection of the orientation of the ellipsoid and rotation of the data points to make the circular structure perpendicular to the fitting coordinate for an upper ellipsoid (A) and for a lower ellipsoid (B). (C) Error estimation as a function of the index $\alpha$.
