# Supplementary figures and images for: Exploring the mechanical and morphological rationality of tree branch structure based on 3D point cloud analysis and the finite element method
Source: Sci Rep. 2022 Mar 8;12:4054. doi: 10.1038/s41598-022-08030-5 (PMC8904476; doi:10.1038/s41598-022-08030-5)

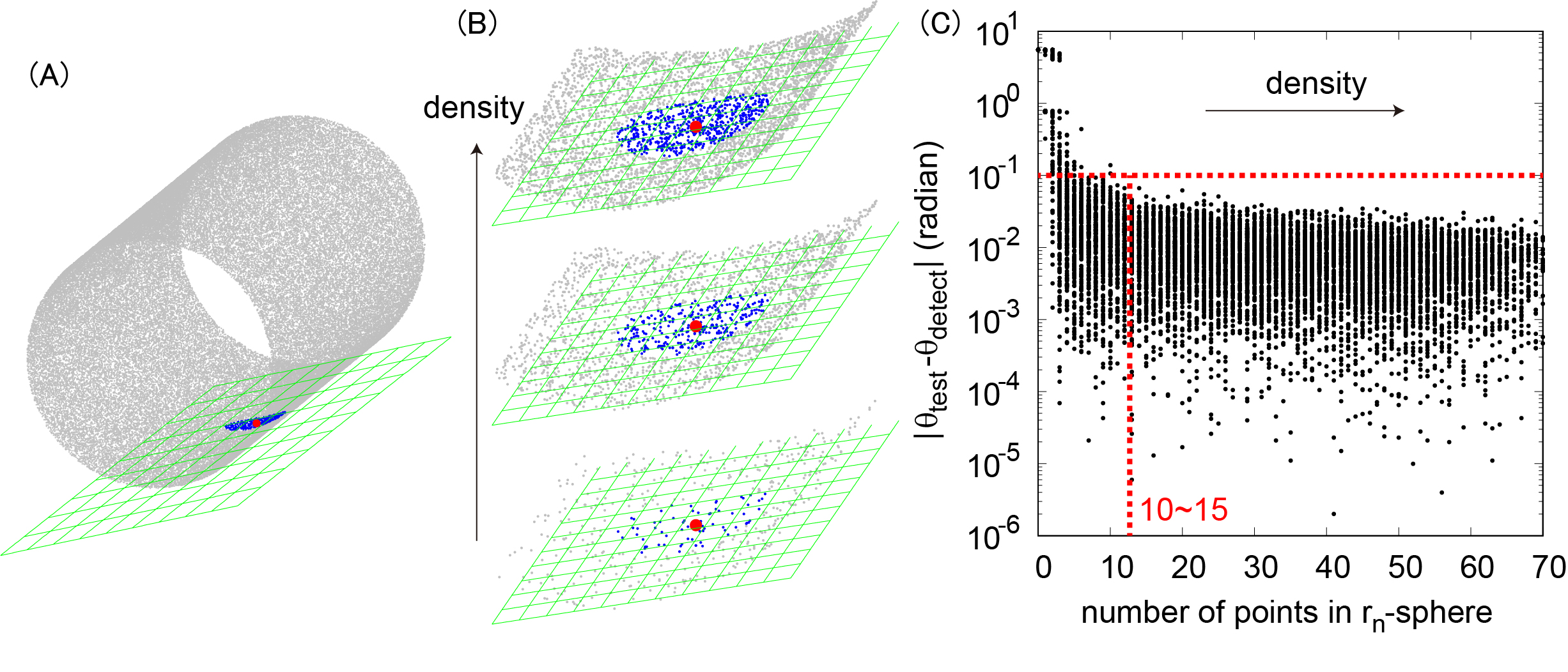

Supplement: Supplementary file 2 — Supplementary Figure 1. [file 41598_2022_8030_MOESM2_ESM.jpg]

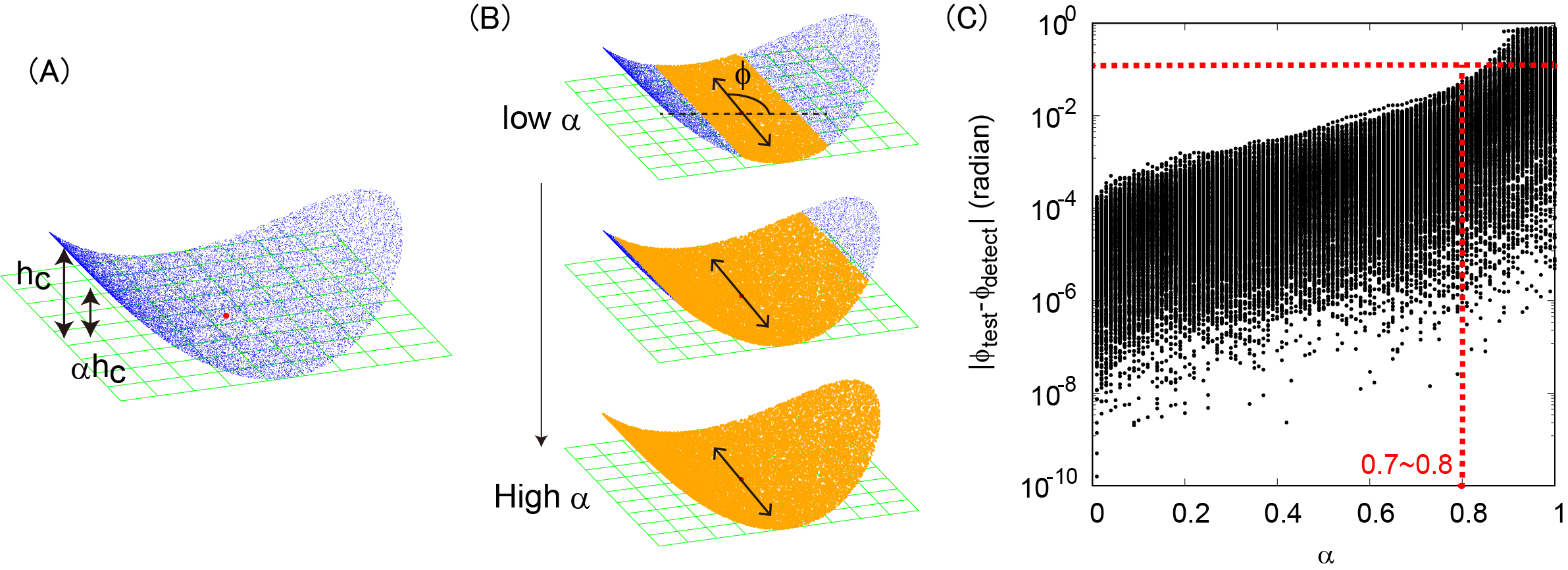

Supplement: Supplementary file 3 — Supplementary Figure 2. [file 41598_2022_8030_MOESM3_ESM.jpg]

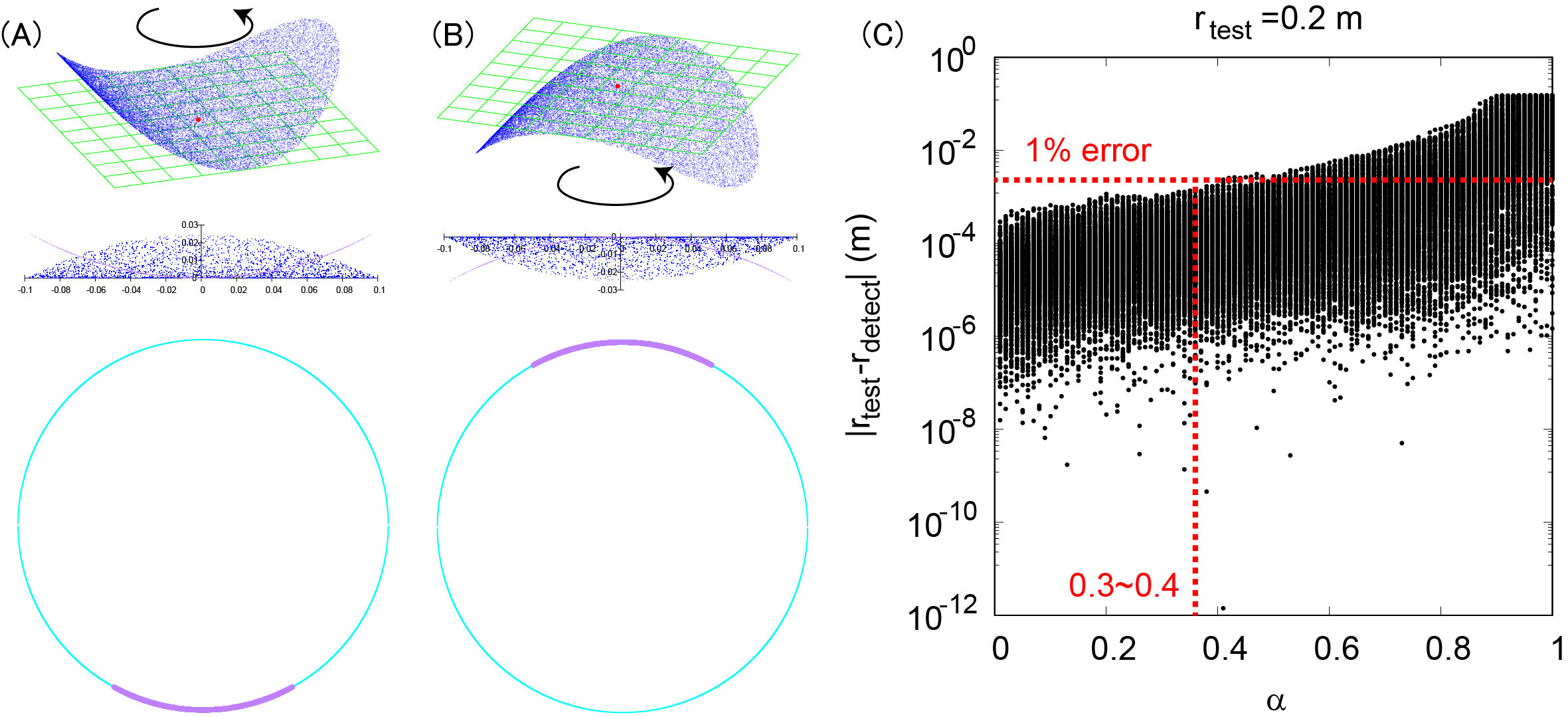

Supplement: Supplementary file 4 — Supplementary Figure 3. [file 41598_2022_8030_MOESM4_ESM.jpg]
